# The Paradox of Copper Deficiency in Wilson's Disease Patients

**DOI:** 10.1002/mdc3.13817

**Published:** 2023-06-30

**Authors:** Raffaele Iorio, Fabiola Di Dato, Maria Immacolata Spagnuolo

**Affiliations:** ^1^ Department of Translational Medical Science University of Naples Federico II Napoli Italy

**Keywords:** Zinc, spinal cord, cytopenia, adverse effect

The paradox of copper deficiency (CD) in patients with Wilson's disease (WD), whose pathogenic hallmark is copper accumulation, is certainly attractive as a possible expression of overtreatment. It is also paradoxical that this deficiency develops in patients treated with zinc rather than in those treated with chelators, which have a stronger action in decoppering the organism. Based on the Chevalier et al's^1^ series and the literature review, therapy‐induced CD in WD patients seems to be a rare event, which, however, must be kept in mind by clinicians managing WD because the clinical and laboratory signs of CD can be overlooked and because they can be erroneously attributed to other causes. Furthermore, it is clinically relevant that, differently from cytopenia because of CD, neurological symptoms induced by CD (especially those of spinal cord involvement) tend to persist despite treatment of CD. Therefore, in the management of WD patients it is greatly desirable to avoid this adverse event.

Copper deficiency can be considered an exaggeration of the decoppering effect, which is the goal of pharmacological treatment of WD. A first question to address is that of the real possibility of decoppering the WD patients with the current therapies. In the real world, a true copper depletion is difficult to obtain if we consider that the studies that have evaluated the concentration of hepatic copper in serial liver biopsies have shown that the copper remains elevated, even in patients with a favorable clinical evolution.^2^, ^3^ Therefore, how can these two phenomena coexist: on the one hand, the persistence of copper accumulation in the liver of wilsonian patients favorably treated and, on the other hand, the risk that the therapy causes an abnormal CD in a small subgroup of them? Because liver biopsy is currently performed infrequently in WD patients both at diagnosis and during follow‐up, it is not well known how hepatic copper accumulation behaves in WD patients with CD due to therapy. Given that the phenomenon of copper deficiency is mainly observed during zinc therapy, whose main mechanism of action is the reduction of intestinal copper absorption and the stabilization of hepatic copper through binding with metallothioneins with consequent reduction of the free toxic fraction, it could be hypothesized that a certain accumulation of copper in the liver may still be present even in the case of CD. In this context, it would certainly be useful to have information on the behavior of the Kayser‐Fleischer ring in patients with CD because Kayser‐Fleischer ring disappearance in WD patients well treated is well documented.^4^, ^5^

Copper deficiency manifests mainly with cytopenia affecting one or more of three blood lineages and spinal cord changes on magnetic resonance imaging. Unfortunately, cytopenias are almost common in patients with WD and may be because of multiple factors for which the role of CD could be questioned, but reversion with therapy modulation and copper supplementation effectively reinforces the pathogenic role of CD. Moreover, given the role of copper in the hematopoiesis process (ceruloplasmin and hephaestin are copper‐dependent ferroxidases required for the export and absorption of iron and synthesis of heme) and the impact of CD in reducing the lifespan of red cells, it is not surprising that a CD can lead to abnormal blood counts. Less easily explained is why CD occurs in only a small subset of cases. In Chevalier et al's^1^ study a patient with CD received, for unexplained reasons, a much higher dose of zinc sulfate than is usually recommended, but in other cases zinc doses were adequate. Unfortunately, however, any predictive factors of CD have been identified.

The study of Chevalier et al^1^ also raises the question of how to monitor the status of body deposits of copper and those of zinc in WD patients on therapy. It is widely accepted that during zinc therapy, the levels of zincuria must be higher than 2000 μg (61 μmol)/24 hours, whereas the cupruria must be lower than 75 μg (1.2 μmol)/24 hours.^4^, ^5^ However, a maximum zinc urinary threshold is not indicated, which could be indicative of overtreatment. Recently, increased attention has been focused on the minimum levels of cupruria below which one must not go down to avoid CD. In recent American Association for the Study of Liver Diseases guidelines, it is reported that the development of cytopenias, an increase in serum ferritin, and/or disproportionately low 24‐hour urinary copper excretion (<100 μg/24 hour for chelation therapy; <20 μg/24 hour [<0.3 μmol/24 hour] for zinc therapy) may be indicative of overtreatment.^4^ Information on the behavior of ferritin is lacking in the Chevalier et al's^1^ study and in the literature review. This study once again underlines the role of free copper determination in monitoring a WD patient under therapy, but as is well known, despite the initial encouraging studies, it is not a test for routine use.^4^

The risk of copper deficiency, therefore, adds to the other possible side effects of zinc therapy such as gastrointestinal disorders and pancreatic hyperenzymemia. Chelators that potentially have more severe adverse effects (paradoxical worsening of neurological symptoms, sensitivity reactions, bone marrow depression, nefropathy, etc) do not appear to induce copper deficiency. In conclusion, although the available drugs have allowed great successes in the treatment of WD, the risk of adverse effects must never be overlooked, therefore, confirming the proposition that any drug can be poison.

## Author Roles

(1) Research project: A. Conception, B. Organization, C. Execution; (2) Statistical Analysis: A. Design, B. Execution, C. Review and Critique; (3) Manuscript Preparation: A. Writing of the First Draft, B. Review and Critique.

R.I.: 1A, 1B, 1C, 3A, 3B, 3C

F.D.D.: 1A, 1B, 1C, 3A, 3B, 3C

M.I.S.: 1A, 1B, 1C, 3A, 3B, 3C

## Disclosures

**Ethical Compliance Statement:** This work does not require the approval of an institutional review board or informed patient consent. We confirm that we have read the Journal's position on issues involved in ethical publication and affirm that this work is consistent with those guidelines.

**Funding Sources and Conflicts of Interest:** No specific funding was received for this work. The authors declare that there are no conflicts of interest relevant to this work.

**Financial Disclosures for Previous 12 Months:** The authors declare that there are no additional disclosures to report.
